# Neuronal overexpression of human TDP-43 in *Caenorhabditis elegans* causes a range of sensorimotor phenotypes

**DOI:** 10.17912/micropub.biology.000766

**Published:** 2023-04-19

**Authors:** Mandy Koopman, Lale Güngördü, Renée I. Seinstra, Ellen A.A. Nollen

**Affiliations:** 1 European Research Institute for the Biology of Ageing, University of Groningen, University Medical Centre Groningen, Laboratory of Molecular Neurobiology of Ageing, The Netherlands

**Figure 1. Graphical summary:**
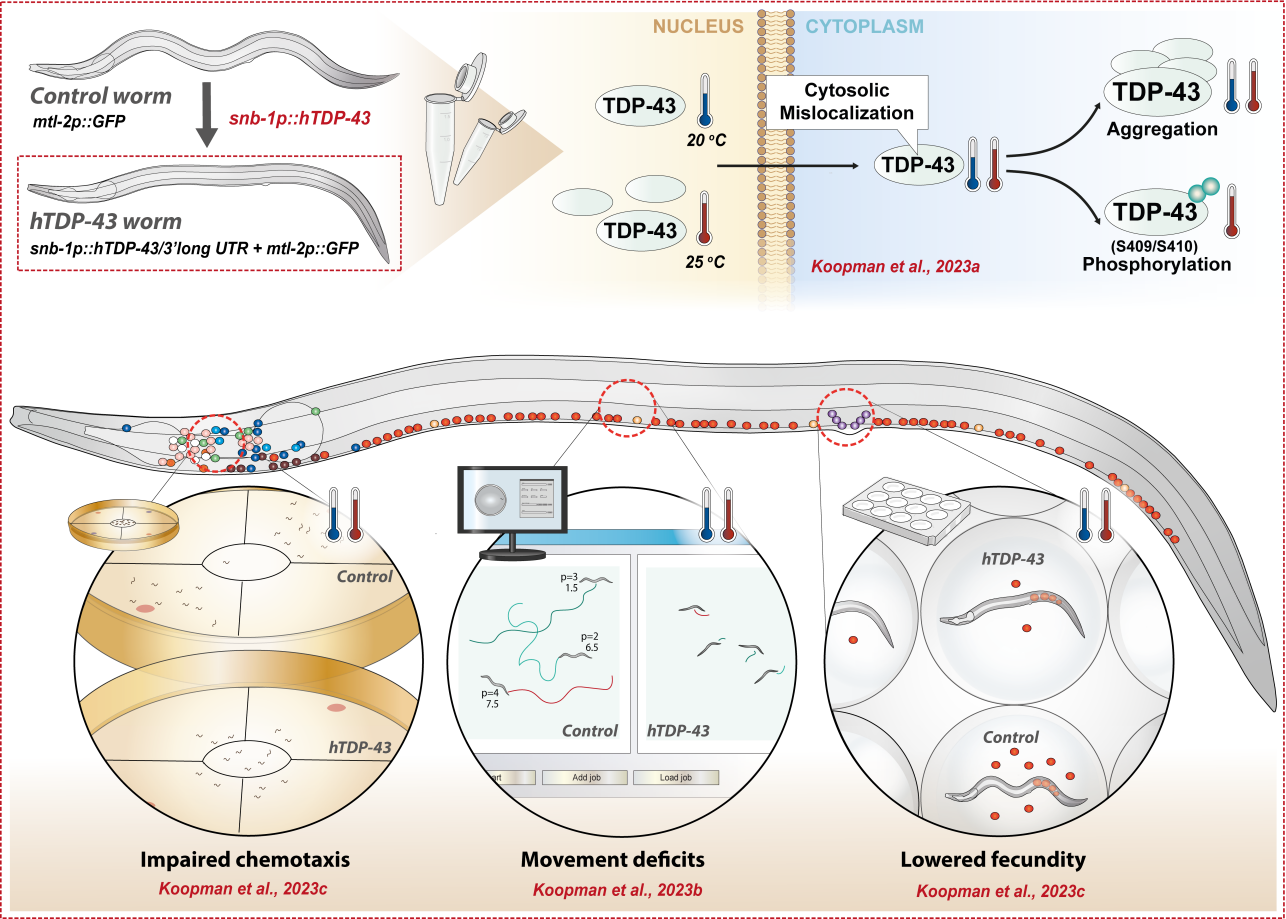
The graphical summary shows the main findings in our series of integrated micropublications. The thermometers illustrate which phenotypic abnormalities are observed at the the different growth temperatures (20 °C or 25 °C).

## Description

Inclusions consisting of transactive response DNA-binding protein 43 (TDP-43) are a characteristic and toxic feature of numerous neurodegenerative diseases, including amyotrophic lateral sclerosis (ALS) (Arai et al., 2006; Neumann et al., 2006; Neumann et al., 2009) . The small nematode *Caenorhabditis elegans* has been instrumental in studying the underlying mechanisms of TDP-43 toxicity *in vivo (* Ash et al., 2010; Liachko et al., 2010; Zhang et al., 2011; Vaccaro et al., 2012; Liachko et al., 2014; Liachko et al., 2019). Previous studies in *C. elegans* models observed the characteristic features of mutant TDP-43 such as; post-translation modifications, insolubility and cytosolic localization. Models expressing wild-type TDP-43, however, either did not show these features or showed less pronounced phenotypes (Ash et al., 2010; Liachko et al., 2010; Vaccaro et al., 2012) . Since wild-type TDP-43 causes lower levels of proteotoxicity than its mutant forms, it has been hypothesized that the extend of locomotor dysfunction correlates with susceptibility of TDP-43 to misfolding, aggregation and phosphorylation in *C. elegans* (Liachko et al., 2010; Vaccaro et al., 2012) . In our series of micropublications we extended the phenotypic landscape of a *C. elegans* model for pan-neuronal expression of hTDP-43. We show that altering the thermal environment of hTDP-43 worms enhances characteristic features of TDP-43 proteinopathies, including increased protein levels, cytosolic localization, and phosphorylation (Koopman et al., 2023a). The proteotoxic effects of hTDP-43 translate into numerous movement phenotypes (Koopman et al., 2023b), disrupted sensorimotor functions and decreased fecundity (Koopman et al., 2023c). Our results suggest that the toxicity of wild-type hTDP-43 in *C. elegans* exceeds the neuromuscular system and affects non-motor systems to a varying degree. Moreover, characteristic *C. elegans* phenotypes, such as coiling, shrinking and uncoordinated movement, may point at defects in specific neuronal subgroups.

**The thermal environment enhances pathological features of wild-type hTDP-43**

Our findings show that raising the growth temperature of hTDP-43 worms enhances numerous features of TDP-43 pathology (Koopman et al., 2023a). The increased phosphorylation of S409/S410 and cytosolic localization of wild-type hTDP-43 are considered pathological hallmarks of TDP-43 proteinopathies. Previous studies did not readily observe cytosolic localized TDP-43 in *C. elegans* (Ash et al., 2010) . Moreover, phosphorylation of TDP-43 is considered highly characteristic for mutant TDP-43 and has not been detected previously when assessing wild-type TDP-43 in *C. elegans* (Liachko et al., 2010; Liachko et al., 2014) . Nonetheless, wild-type TDP-43 harbors the propensity to undergo post-translation modifications and form insoluble aggregates (Arai et al., 2006; Neumann et al., 2006; Neumann et al., 2009) . Here, we show that by altering the environmental context, e.g., the growth temperature, such features can be detected in *C. elegans* (Koopman et al., 2023a).

Whether our observations represent actual TDP-43 pathology (e.g., misfolding, mislocalization), as previously postulated by Zhang et al. (Zhang et al., 2011) , or merely mirrors the increased expression of hTDP-43 at higher growth temperatures remains to be determined. The synaptobrevin promotor used to drive pan-neuronal expression of hTDP-43 clearly acts as a temperature-sensitive regulator of expression and thus enhances the expression of the hTDP-43 at higher temperatures. It is however, clear that cytosolic localization of hTDP-43 is increased in both absolute and relative numbers when comparing hTDP-43 worms grown at 25 °C to those raised at 20 °C (Koopman et al., 2023a). Therefore, it is likely that additional effectors are at play besides the titrating effect of the environmental temperature on hTDP-43 expression levels.

**TDP-43 worms show phenotypes associated with defects in cholinergic and GABAergic signaling**

Movement dysfunction is a characteristic phenotype of worms overexpressing (mutant) hTDP-43 (Ash et al., 2010; Liachko et al.,2010). In line with previous studies, we show that neuronal expression of hTDP-43 in *C. elegans* severely impairs spontaneous and induced movement (Koopman et al., 2023b). Since we assayed locomotion and body movement to a new extent, our results add another dimension to the previously described phenotypes in liquid- and on solid medium. For example, hTDP-43 worms are not only uncoordinated but also show context-dependent shrinking and coiling, features that are seen in worms with cholinergic and GABAergic defects (Rand et al., 1984; Schuske et al., 2004; Sluder et al., 2012) .

So far, GABAergic dysfunction and degeneration have been considered a specific consequence of neuronal expression of mutant hTDP-43 (Vaccaro et al., 2012) . Expression of wild-type hTDP-43 only results in small but significant alterations in the distribution and number of GABAergic synapses (Ash et al., 2010) . Cholinergic transmission, on the other hand, has received little attention in previous studies. Our results suggest that a detailed (functional) dissection of the cholinergic system may provide additional insights in the neurotoxic mechanisms of hTDP-43.

**Widespread proteotoxicity of hTDP-43 in extra-motor systems**

Neuronal circuits integrate various environmental stimuli to alter behavior, including locomotory patterns(Bargmann et al., 1991; Sawin et al.,2000). Evaluating the behavior of nematodes in the presence of multiple environmental cues provides a way to analyze different components of the nervous system that influence motor output. Both serotonin and dopamine are known to modulate the output of motoneurons (Nurrish et al., 1999; Sawin et al., 2000; Chase et al., 2004) . Serotonin inhibits acetylcholine release via activation of the *goa-1* Gα _o_ mediated-pathway (Nurrish et al., 1999) while dopamine’s effects are dependent on the ratio of DOP-1/DOP-3 expression in motoneurons (Chase et al., 2004) . Chemotaxis is mediated by glutamatergic sensory neurons(De Bono et al., 2005; Ohnishi et al., 2011) that synapse on a dense network of interneurons that eventually provide output to motoneurons via the command motoneurons AVA and AVB (Piggott et al., 2011; Fang-Yen et al., 2015) .

We show that olfactory-driven movement is abrogated in hTDP-43 worms while the mechanosensory, dopamine-regulated basal slowing response is seemingly intact (Koopman et al., 2023c). Moreover, we find that hTDP-43 worms have lower fecundity and might have a higher basal egg-laying rate in M9 (Koopman et al., 2023c). There is, however, no clear response to 5-HT treatment (Koopman et al., 2023c). The reduction in offspring as a consequence of hTDP-43 expression has been previously described (Zhang et al., 2012) . Our results, however, also contradict earlier observations showing that most offspring of hTDP-43 worms die during embryogenesis when exposed to an environmental temperature of 25 °C (Zhang et al., 2012) . While we initially observed similar effects, we did not observe embryogenic lethality after six additional backcrosses. This could be the result of additional background mutations, transgenic silencing or adaptation over time by the accumulation of suppressor mutations.

Our findings do not exclude altered serotonergic or dopaminergic signalling in hTDP-43 worms (Koopman et al., 2023c). Both the (non-significant, but underpowered) increase in basal egg-laying rate and the reduced effect of food on movement speed hint towards increased serotonergic and dopaminergic signalling respectively as both transmitters can negatively influence movement through the inhibition of cholinergic motoneurons (Nurrish et al., 1999) . Nonetheless, due to severe movement defects in hTDP-43 worms, differences in food-slowing might as well be the consequence of the reduced response-window (i.e. absolute speed cannot be lower than 0). Therefore, additional studies should further elucidate whether sensory, dopaminergic, and serotonergic systems are contributing to the observed movement dysfunction in hTDP-43 worms or represent epiphenomena.

**The interaction effects of increased hTDP-43 levels and accelerated aging**

An increased environmental temperature enhanced most, but not all phenotypic abnormalities in hTDP-43 worms. A higher growth temperature is known to have a major effect on the physiology of the worms since it affects both the rate of development and rate of aging (Klass et al., 1977; Lee and Kenyon, 2009) . While worms grown at 20 °C and 25 °C were examined at the same chronological time, they consequently had a different biological age. In case of hTDP-43 worms, temperature also enhanced hTDP-43 levels and accompanying pathological features. Therefore, it is impossible to separate the accelerated aging effects from the potential effects of increased hTDP-43 levels on the measured phenotypic parameters. In fact, given the similar effects of environmental temperature on locomotion capacity of control and hTDP-43 worms, the biological age may account for a large part of the temperature-evoked phenotypic response (Koopman et al., 2023a; Koopman et al., 2023b; Koopman et al., 2023c).

The inability to separate aging and hTDP-43 specific effects does not preclude the use of temperature to increase the severity of movement dysfunction in hTDP-43 worms for screening purposes. An environmental temperature of 25 °C can be used to completely abolish the thrashing capacity of hTDP-43 worms. Consequently, within thrashing frequency of hTDP-43 worms resides a robust and binary readout that can be used in elegant suppressor screens. At the same time other behaviors that require basal locomotion, like chemotaxis or food-slowing, become inaccessible at 25 °C. Thus, the usefulness of an increased growth temperature highly depends on the feature to be assessed and the question to be answered. Altogether, these findings reinforce the idea that environmental factors can be used to manipulate the genotype-phenotype conversion and, ergo, to shape screenable phenotypes.

## Reagents

**Table 1:** Strains used

**Table:** 

**Strain**	**Description**	**Genotype**	**Remark**
N2	Bristol wild isolate	Wildtype	
OW1601	CL6049 6x backcrossed with N2 . Named: hTDP-43 worms.	*dvIs62* [ *snb-1p::hTDP-43/3’long UTR + mtl-2p::GFP* ]X	Provided by Chris Link
OW1603	CL2122 6x backcrossed with N2 . Named: control worms.	*dvIs15* [( *pPD30.38) unc-54 (vector) + (pCL26)mtl-2p::GFP* ]	Provided by Chris Link

## References

[R1] Arai T, Hasegawa M, Akiyama H, Ikeda K, Nonaka T, Mori H, et al., Oda T. 2006. TDP-43 is a component of ubiquitin-positive tau-negative inclusions in frontotemporal lobar degeneration and amyotrophic lateral sclerosis. Biochem Biophys Res Commun 351: 602-11. 10.1016/j.bbrc.2006.10.093 17084815

[R2] Ash PE, Zhang YJ, Roberts CM, Saldi T, Hutter H, Buratti E, Petrucelli L, Link CD. 2010. Neurotoxic effects of TDP-43 overexpression in C. elegans. Hum Mol Genet 19: 3206-18. 10.1093/hmg/ddq230 PMC2908471 20530643

[R3] Bargmann CI, Horvitz HR (1991). Chemosensory neurons with overlapping functions direct chemotaxis to multiple chemicals in C. elegans.. Neuron.

[R4] Chase DL, Pepper JS, Koelle MR (2004). Mechanism of extrasynaptic dopamine signaling in Caenorhabditis elegans.. Nat Neurosci.

[R5] de Bono M, Maricq AV (2005). Neuronal substrates of complex behaviors in C. elegans.. Annu Rev Neurosci.

[R6] Fang-Yen C, Alkema MJ, Samuel AD. 2015. Illuminating neural circuits and behaviour in Caenorhabditis elegans with optogenetics. Philos Trans R Soc Lond B Biol Sci 370: 20140212. 10.1098/rstb.2014.0212 PMC4528824 26240427

[R7] Klass MR. 1977. Aging in the nematode Caenorhabditis elegans: major biological and environmental factors influencing life span. Mech Ageing Dev 6: 413-29. 10.1016/0047-6374(77)90043-4 926867

[R8] Koopman M, Güngördü L, Seinstra RI, Hogewerf W & Nollen EAA. 2023a. Neuronal overexpression of hTDP-43 in *C. elegans* mimics the cellular pathology commonly observed in TDP-43 proteinopathies. microPublication Biology 10.17912/micropub.biology.000767 PMC10163326 37159575

[R9] Koopman M, Güngördü L, Seinstra RI & Nollen EAA. 2023b. Neuronal overexpression of hTDP-43 in *C. elegans* impair motor function. microPublication Biology 10.17912/micropub.biology.000768 PMC10163325 37159576

[R10] Koopman M, Güngördü L, Seinstra RI & Nollen EAA. 2023c. Neuronal overexpression of hTDP-43 in *C. elegans* impairs different neuronally controlled behaviors and decreases fecundity. microPublication Biology 10.17912/micropub.biology.000769 PMC10157382 37151215

[R11] Lee SJ, Kenyon C (2009). Regulation of the longevity response to temperature by thermosensory neurons in Caenorhabditis elegans.. Curr Biol.

[R12] Liachko NF, Saxton AD, McMillan PJ, Strovas TJ, Keene CD, Bird TD, Kraemer BC. 2019. Genome wide analysis reveals heparan sulfate epimerase modulates TDP-43 proteinopathy. PLoS Genet 15: e1008526. 10.1371/journal.pgen.1008526 PMC6934317 31834878

[R13] Liachko NF, Guthrie CR, Kraemer BC (2010). Phosphorylation promotes neurotoxicity in a Caenorhabditis elegans model of TDP-43 proteinopathy.. J Neurosci.

[R14] Liachko NF, McMillan PJ, Strovas TJ, Loomis E, Greenup L, Murrell JR, et al., Kraemer BC. 2014. The tau tubulin kinases TTBK1/2 promote accumulation of pathological TDP-43. PLoS Genet 10: e1004803. 10.1371/journal.pgen.1004803 PMC4256087 25473830

[R15] Neumann M, Kwong LK, Lee EB, Kremmer E, Flatley A, Xu Y, et al., Lee VM. 2009. Phosphorylation of S409/410 of TDP-43 is a consistent feature in all sporadic and familial forms of TDP-43 proteinopathies. Acta Neuropathol 117: 137-49. 10.1007/s00401-008-0477-9 PMC2693625 19125255

[R16] Neumann M, Sampathu DM, Kwong LK, Truax AC, Micsenyi MC, Chou TT, Bruce J, Schuck T, Grossman M, Clark CM, McCluskey LF, Miller BL, Masliah E, Mackenzie IR, Feldman H, Feiden W, Kretzschmar HA, Trojanowski JQ, Lee VM (2006). Ubiquitinated TDP-43 in frontotemporal lobar degeneration and amyotrophic lateral sclerosis.. Science.

[R17] Nurrish S, Ségalat L, Kaplan JM (1999). Serotonin inhibition of synaptic transmission: Galpha(0) decreases the abundance of UNC-13 at release sites.. Neuron.

[R18] Ohnishi N, Kuhara A, Nakamura F, Okochi Y, Mori I. 2011. Bidirectional regulation of thermotaxis by glutamate transmissions in Caenorhabditis elegans. EMBO J 30: 1376-88. 10.1038/emboj.2011.13 PMC3094115 21304490

[R19] Piggott BJ, Liu J, Feng Z, Wescott SA, Xu XZ. 2011. The neural circuits and synaptic mechanisms underlying motor initiation in C. elegans. Cell 147: 922-33. 10.1016/j.cell.2011.08.053 PMC3233480 22078887

[R20] Rand JB, Russell RL. 1984. Choline acetyltransferase-deficient mutants of the nematode Caenorhabditis elegans. Genetics 106: 227-48. 10.1093/genetics/106.2.227 PMC1202253 6698395

[R21] Sawin ER, Ranganathan R, Horvitz HR. 2000. C. elegans locomotory rate is modulated by the environment through a dopaminergic pathway and by experience through a serotonergic pathway. Neuron 26: 619-31. 10.1016/s0896-6273(00)81199-x 10896158

[R22] Schuske K, Beg AA, Jorgensen EM. 2004. The GABA nervous system in C. elegans. Trends Neurosci 27: 407-14. 10.1016/j.tins.2004.05.005 15219740

[R23] Sluder A, Shah S, Cassayre J, Clover R, Maienfisch P, Molleyres LP, Hirst EA, Flemming AJ, Shi M, Cutler P, Stanger C, Roberts RS, Hughes DJ, Flury T, Robinson MP, Hillesheim E, Pitterna T, Cederbaum F, Worthington PA, Crossthwaite AJ, Windass JD, Currie RA, Earley FG (2012). Spiroindolines identify the vesicular acetylcholine transporter as a novel target for insecticide action.. PLoS One.

[R24] Vaccaro A, Tauffenberger A, Aggad D, Rouleau G, Drapeau P, Parker JA (2012). Mutant TDP-43 and FUS cause age-dependent paralysis and neurodegeneration in C. elegans.. PLoS One.

[R25] Zhang T, Hwang HY, Hao H, Talbot C Jr, Wang J (2012). Caenorhabditis elegans RNA-processing protein TDP-1 regulates protein homeostasis and life span.. J Biol Chem.

[R26] Zhang T, Mullane PC, Periz G, Wang J (2011). TDP-43 neurotoxicity and protein aggregation modulated by heat shock factor and insulin/IGF-1 signaling.. Hum Mol Genet.

